# New parameters describing morphological variations in the suprascapular notch region as potential predictors of suprascapular nerve entrapment

**DOI:** 10.1186/1471-2474-15-396

**Published:** 2014-11-25

**Authors:** Michał Podgórski, Mirosław Topol, Marcin Sibiński, Piotr Grzelak, Ludomir Stefańczyk, Michał Polguj

**Affiliations:** Department of Angiology, Chair of Anatomy, Medical University of Łódź, Narutowicza 60, Łódź, 90-136 Poland; Department of Normal and Clinical Anatomy, Chair of Anatomy, Medical University of Łódź, Łódź, Poland; Clinic of Orthopaedic and Pediatric Orthopaedics, Medical University of Łódź, Łódź, Poland; Department of Radiology, Medical University of Łódź, Łódź, Poland

**Keywords:** Suprascapular nerve entrapment, Superior transverse scapular ligament, Suprascapular notch, Suprascapular foramen

## Abstract

**Background:**

The suprascapular notch (SSN), bridged by the superior transverse scapular ligament (STSL), creates a pathway for the suprascapular nerve (SN). Morphological variations in the SSN region are common and can increase the risk of neuropathy by constricting the space for the nerve. The aim of this study was to establish new objective parameters that take this complex morphology into account.

**Methods:**

The SSN region of 100 formalin-fixed cadaveric shoulders was dissected. The dimensions of the SSN, the STSL and the anterior coracoscapular ligament (ACSL), as well as diameters of the SN, associated vessels and SN passage area, were measured by means of quantitative visual data analysis software to assign those structures to present classifications. The area reduction coefficient (ARC) and the ambit occupation coefficient (AOC) were defined and calculated for each shoulder.

**Results:**

The mean ARC and AOC for ligaments in the suprascapular region were: ARC_STSL_ = 71.6%, ARC_ACSL_ = 9.6%, AOC_STSL_ = 56.8% and AOC_ACSL_ = 9.1%. The SN passage area, ARC and AOC did not differ significantly between SSN types. The SN passage area and ARC differed significantly between band- and fan-shaped types of STSL. A significant relationship was observed between ARC and AOC (R = 0.6855; p < 0.0001). The SN passage area correlated significantly with ARC (R = -0.7555; p < 0.0001) and AOC (R = -0.5609; p < 0.0001).

**Conclusions:**

The proposed parameters convey the complex morphology of the SSN region in a quantitative manner. The area reduction coefficient seems to be a more relevant indicator than the AOC as it better correlates with the SN passage area. Contrary to the SSN type, the STSL type significantly influences SN passage area and ARC.

**Electronic supplementary material:**

The online version of this article (doi:10.1186/1471-2474-15-396) contains supplementary material, which is available to authorized users.

## Background

The suprascapular nerve originates predominantly from the ventral rami of the C5 and C6 nerve roots, and after separation from the upper trunk of brachial plexus passes above the upper border of the scapula through an osteo-fibrous tunnel comprising the suprascapular notch (SSN) bridged by the superior traverse scapular ligament (STSL) [1]. It typically travels with the suprascapular vein [1]. The associated artery runs above the STSL [1]. The suprascapular nerve innervates the supraspinatus and infraspinatus muscles and also supports sensory branches to the posterior side of the glenohumeral joint capsule and up to 70% of skin of the shoulder [2]. Compression or injury to the SN can result in SN entrapment syndrome, which was first described in 1936 by André Thomas [3].

Many anatomical variations of the SSN region make it a very heterogeneous structure. The shapes of the SSN and the STSL are highly diverse [4–7], as is the occurrence of the anterior coracoscapular ligament (ACSL), which is reported with a frequency ranging from 18.8% [5] to 60% [8]. A tight and narrow SSN or a broad, bifid or ossified STSL/ACSL can obstruct the SN passage, increasing the risk of its irritation, which may eventually lead to the development of proximal SN entrapment syndrome [2].

The aim of this study is to establish the parameters that relate to reductions in SN passage area and which also take into account the constellation of anatomical variations in the SSN region. Those new parameters may assist quantitative evaluation of the risk of SN neuropathy.

## Methods

The suprascapular notches of 100 formalin-fixed, cadaveric shoulders (41 left and 59 right) were dissected. All shoulders originated from the Polish population. All donors gave informed consent for their bodies to be used for scientific purposes. Ethical approval to undertake this study was obtained from the Bioethics Commission of the Medical University of Lodz (protocol no. RNN/580/13/KE).

The arrangement of the SN and vessels in relation to the STSL was identified. Photographic documentation of structures in the SSN region was obtained. Quantitative image analysis software (MultiScanBase 18.03 software, Computer Scanning System II, Warsaw, Poland) was used to measure the length of the STSL, as well as its proximal and distal width, as well as the maximal depth, superior and middle transverse diameters of the SSN, as described by Polguj et al. [6].

The SSN and STSL were classified into different types according to newest classifications for both of these structures [6, 9]. The SN passage area and the STSL area were evaluated. When present, the area of the ACSL was also evaluated, together with the area of the opening below it.

**Figure 1:**
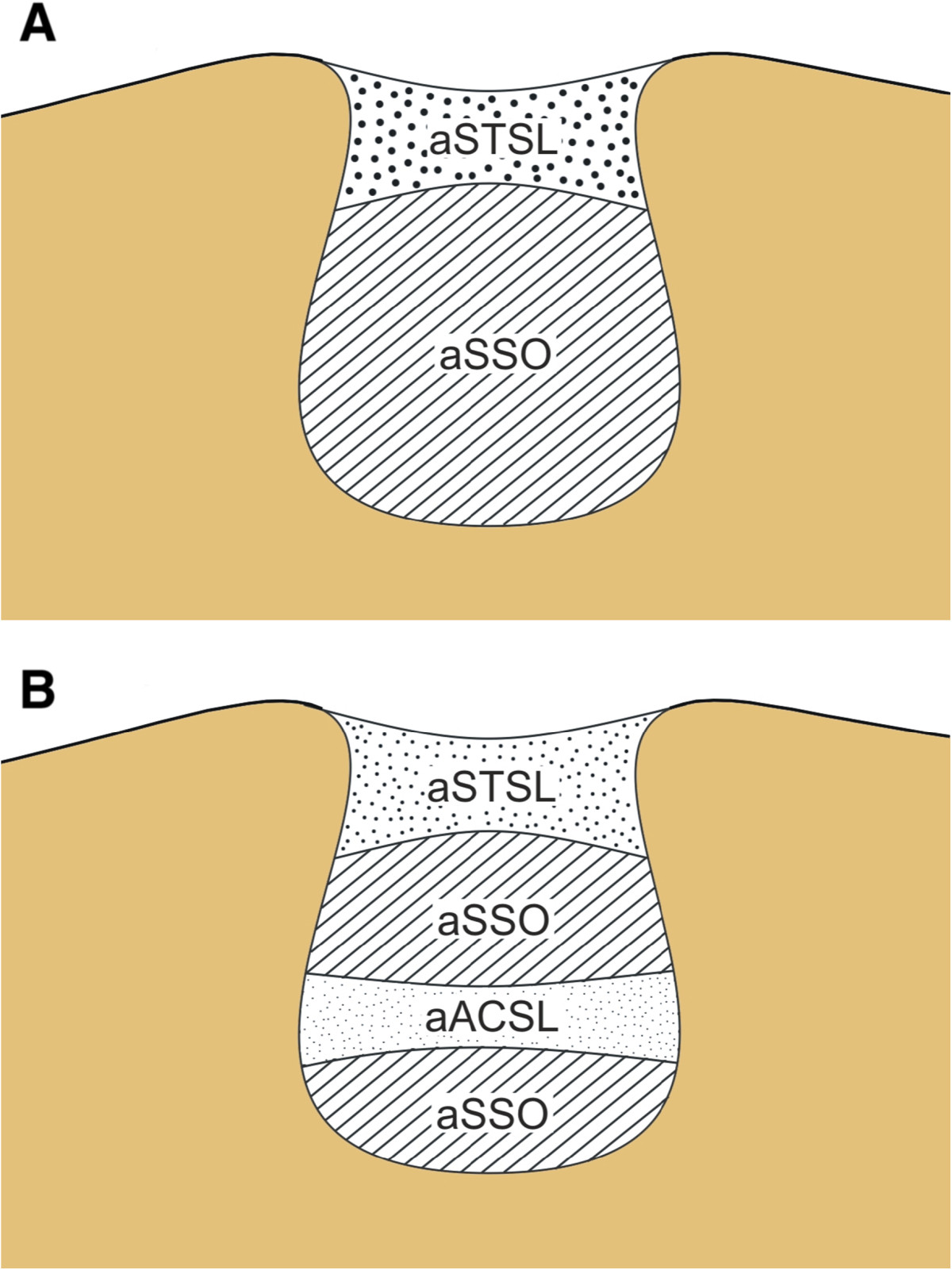
**Schematic representation of the measurements of the areas in the suprascapular region: aSTSL - area of the superior transverse scapular ligament; aACSL - area of the anterior coracoscapular ligament; aSSO - area of the suprascapular opening. A** – specimens without ACSL. **B** – specimens with ACSL.

**Figure 2:**
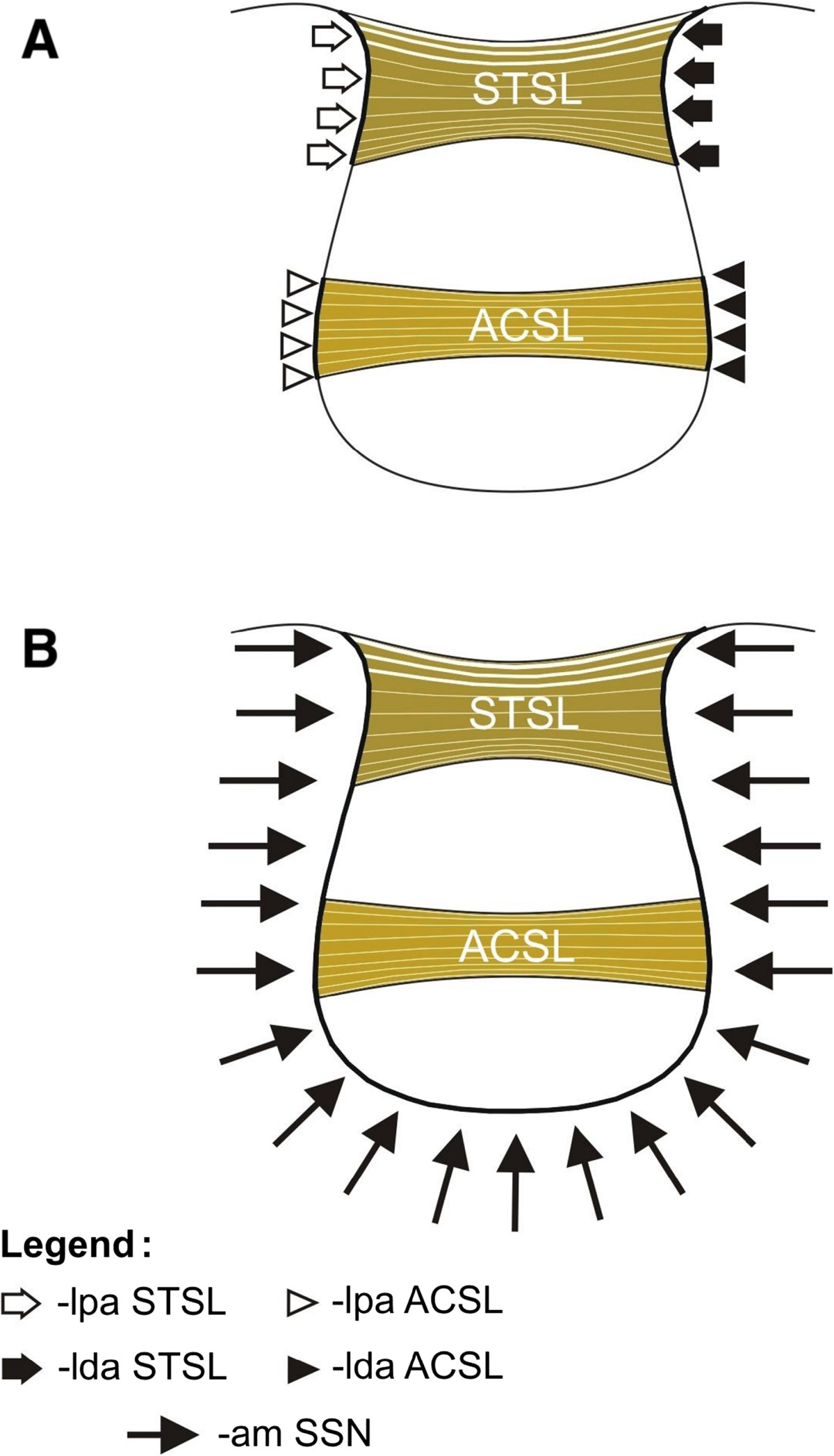
**Schematic representation of the arrangements of the structures in the suprascapular region: amSSN - ambit of the suprascapular notch (B); ldaSTSL - length of the distal attachment of the STSL (A), lpaSTSL - length of the proximal attachment of the STSL (A), lpaACSL - length of the proximal attachment of the ACSL (A), ldaACSL - length of the distal attachment of the ACSL (A).** STSL – superior transverse scapular ligament, ACSL – anterior coracoscapular ligament.

Based on the photographic documentation analysis, two new parameters were defined and described as follows (Figures 1 and 2):

1. The area reduction coefficient (ARC), calculated according to the following formula (Figure 1):
  $$\mathrm{ARC}\;\left[\%\right]\;=\;ARC_{\mathrm{STSL}}\left[\%\right]\;+\;ARC_{\mathrm{ACSL}}\left[\%\right]*$$

* when ACSL is present

- Area reduction coefficient of the STSL (ARC_STSL_) (Figure 1A):
- Area reduction coefficient of the ACSL (ARC_ACSL_) (Figure 1B):

* when ACSL present

$$ARC_{\mathrm{STSL}}\left[\%\right]\;=\;\left(\mathrm{aSTSL}/\mathrm{aSSN}\right)\;\times \;100$$

$$ARC_{\mathrm{ACSL}}\left[\%\right]\;=\;\left(\mathrm{aACSL}/\mathrm{aSSN}\right)\;\times \;100$$

$$\mathrm{aSSN}=\mathrm{aSTSL}+\mathrm{aACS}L^{*}+\mathrm{aSSO}$$

aSSN - area of the suprascapular notch - area limited by the superior border of the STSL and bony border of the SSN.

aSTSL - area of the superior transverse scapular ligament - area limited by the superior and inferior border of the STSL and bony borders of the SSN at the line of proximal and distal attachment of the STSL.

aACSL - area of the anterior coracoscapular ligament - area limited by the superior and inferior border of the ACSL and bony borders of the SSN at the line of proximal and distal attachment of the ACSL.

aSSO - area of the suprascapular opening - area of the suprascapular notch that is not occupied by the STSL and/or ACSL. When the ACSL is absent, the aSSO is identical with that of the SN passage (Figure 1A). However, when the ACSL is present, it divides the aSSO into the SN passage and the opening between its lower border and bony border of the SSN (Figure 1B).

2. The ambit occupation coefficient (AOC), calculated according to the following formula (Figure 2):
  $$AOC\;\left[\%\right]\;=\;AOC_{\mathrm{STSL}}\left[\%\right]\;+\;AOC_{\mathrm{ACSL}}\left[\%\right]*$$

* when ACSL present

- The ambit occupation coefficient of the STSL (AOC_STSL_)
- The ambit occupation coefficient of the ACSL (AOC_ACSL_)

$$AOC_{\mathrm{STSL}}\left[\%\right]\;=\;\left[\left(\mathrm{ldaSTSL}\;+\mathrm{lpaSTSL}\right)/\mathrm{amSSN}\right]\;\times \;100$$

$$AOC_{\mathrm{ACSL}}\left[\%\right]\;=\;\left[\left(\mathrm{ldaACSL}\;+\;\mathrm{lpaACSL}\right)/\mathrm{amSSN}\right]\;\times \;100$$

amSSN - ambit of the suprascapular notch - length of the bony border of the SSN.

ldaSTSL - length of the distal attachment of the STSL - length of the bony border adjacent to the distal attachment of the STSL.

lpaSTSL - length of the proximal attachment of the STSL - length of the bony border adjacent to the proximal attachment of the STSL.

ldaACSL - length of the distal attachment of the ACSL - length of the bony border adjacent to the distal attachment of the ACSL.

lpaACSL - length of the proximal attachment of the ACSL - length of the bony border adjacent to the distal attachment of the ACSL.

All parameters give information about the contribution of the different types of SSN and STSL, as well as the presence of the ACSL, to the total area reduction of the SN passage. The normality of data distribution was tested with the Shapiro-Wilk test. The difference between the two types of STSL with regard to ARC, AOC and SN passage area was evaluated by the Mann-Whitney test. The comparison of those parameters between SSN types was tested with the Kruskal–Wallis one-way analysis of variance with dedicated post-hoc analysis. Correlations were evaluated by means of the Spearman's rank correlation coefficient. For the statistical analysis, p < 0.05 was considered significant.

## Results

**Figure 3:**
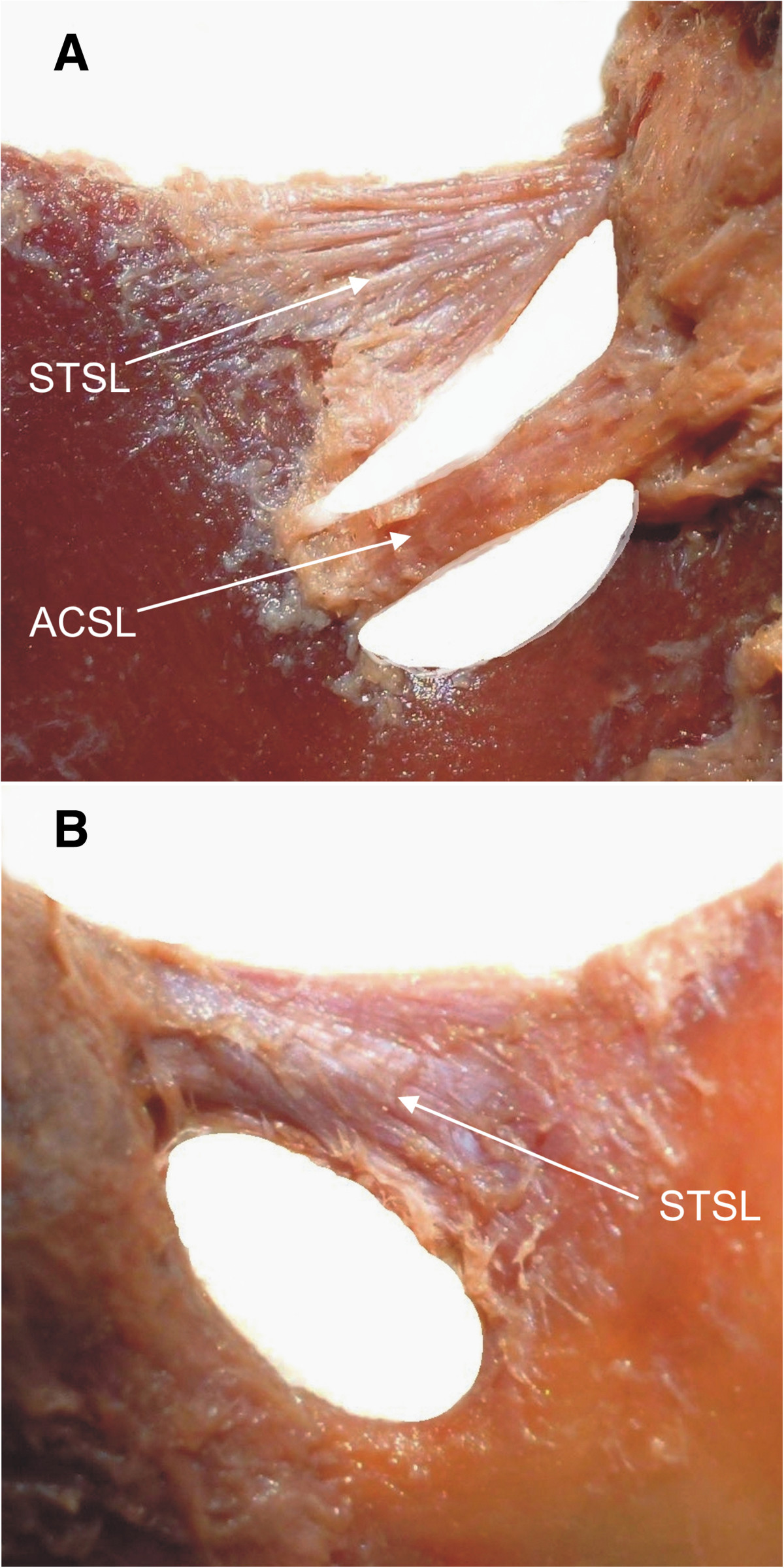
**Structures of the suprascapular region: STSL – superior transverse scapular ligament, ACSL – anterior coracoscapular ligament. A** – fan-shaped STSL. **B** – band-shaped STSL.

In all cases, the SN travelled below the STSL together with the suprascapular vein. The mean cross-sectional area of the suprascapular nerve was found to be 3.43 mm^2^ ± 0.42 mm^2^ (mean ± standard deviation). The mean diameters of the suprascapular artery and vein were 2.17 mm (SD = 0.5 mm) and 3.52 mm (SD = 0.6 mm), respectively. In 4 specimens, the suprascapular artery travelled below the STSL. An ACSL was present in 52 specimens (Figure 3A).

### Area reduction coefficient (ARC)

**Figure 4:**
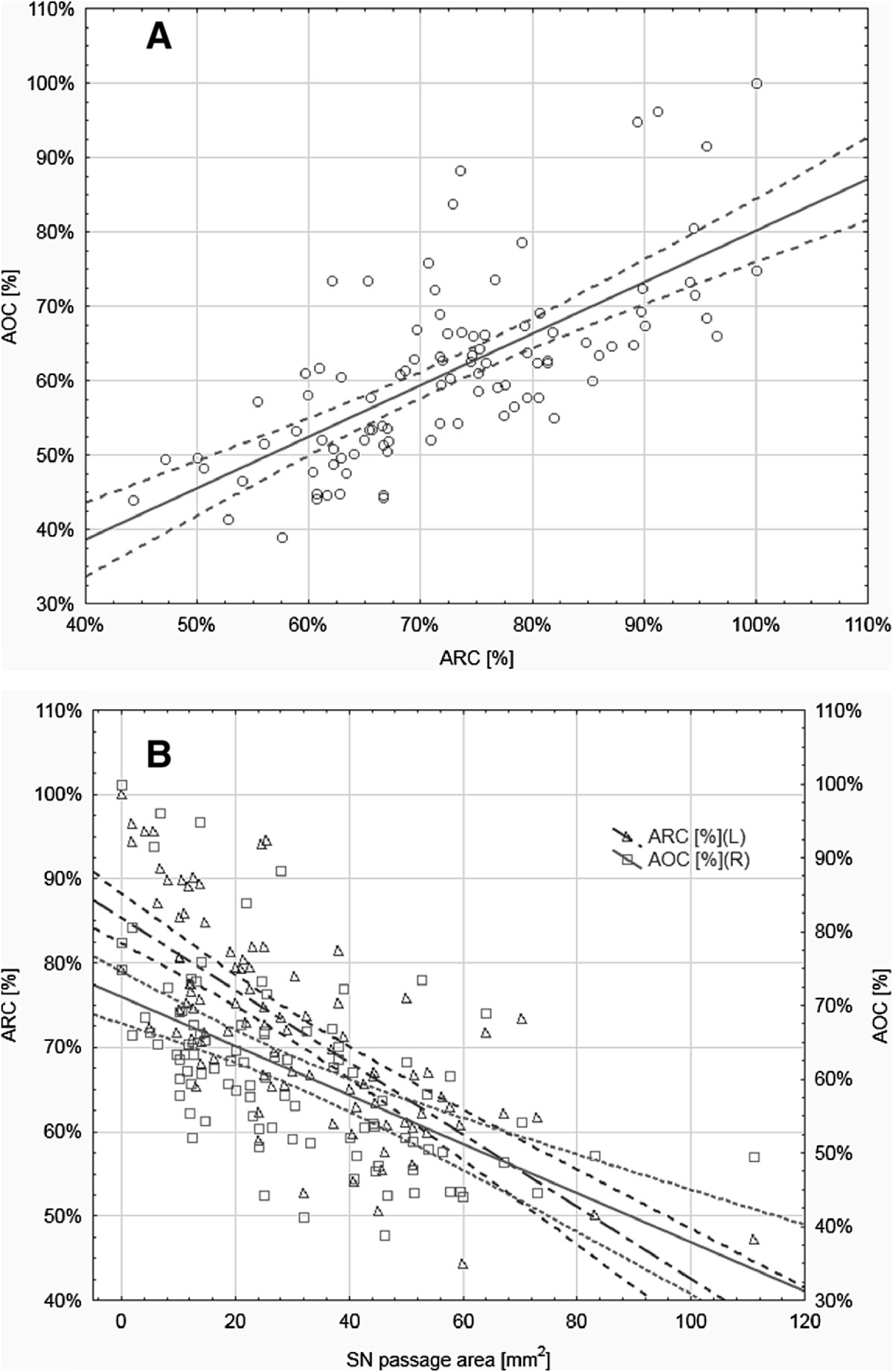
**Correlation plots between area reduction coefficient (ARC) and ambit occupation coefficient (AOC) (A) and between SN passage area and both parameters (B).**

The mean value for the ARC was 72.6% ±12% (mean ± standard deviation), being 71.6% ±26.4%, 9.6% ±7.6% and 32% ±21.1% for the aSTSL, aACSL and aSSO, respectively (Table 1). The area reduction coefficient was significantly correlated with the ambit occupation coefficient (R = 0.6855; p < 0.0001) (Figure 4A) and with SN passage area (R = -0.7555; p < 0.0001) (Figure 4B). The correlation with SN passage area was stronger in band-shaped STSL (R = -0.7064; p < 0.0001) than in the fan-shaped type (R = -0.6347; p < 0.0001). The ARC values for fan-shaped (Figure 3A) and band-shaped (Figure 3B) STSL types were estimated to be 65.3% and 70.9%, respectively (Table 1), which was significantly different (p = 0.0274). Furthermore, ARC_STSL_ correlated inversely with the ARC_ACSL_(R = -0.306; p = 0.0022). This correlation was stronger in band-shaped STSL (R = -0.4753; p = 0.0011) than in the fan-shaped type (R = -0.3155; p = 0.0201). Table 1 presents the ARC values evaluated within different types of SSN and STSL in detail.

**Table 1:** **Characteristics of ARC associated parameters according to STSL and SSN types**

Parameters of area reduction		STSL		SSN type								
		Fan-type	Band-type	IA	IB	IC	II	IIIA	IIIB	IIIC	IV	V
Number		56	44	2	1	2	11	6	13	59	3	3
aSSN [mm^2^]	Mean	119.1	93.66	140.7	240	109.75	100.1	111.4	130.1	103.35	106.71	43.2
	SD	42.3	31.17	76.01	-	7.42	27.7	28.87	47.98	34.02	17.62	29
	p	0.0032		0.0556								
aSTSL [mm^2^]	Mean	76.8	65.3	94.3	172	78.1	67.9	77.6	78.23	68.38	91.37	34.6
	SD	28.12	23	10.3	-	17.68	26.09	13.36	31.55	22.52	6.86	24.23
	p	0.05887		0.0261^†^								
aACSL [mm^2^]*	Mean	9.03	10.12	18.2	-	11.3	11.59	-	11.39	9.2	-	-
	SD	8.69	6.63	-	-	1.84	7.31	-	9.02	7.32	-	-
	p	0.1996		0.3423								
aSSO [mm^2^]	Mean	38.56	23.9	41.95	64	21.85	26.89	32.17	47.21	31.1	13.17	9.1
	SD	22.32	16.33	59.3	-	9.4	14.5	18.97	28.67	17.96	8.58	6.99
	p	0.0004		0.1395								
SN passage area [mm^2^]	Mean	36.84	20.86	36.5	64	7.8	22.78	32.17	43.8	29.5	13.17	7.8
	SD	21.89	15.46	51.2	-	3.96	13.22	18.97	30.6	17.2	8.58	5.35
	p	0.00017		0.0306^†^								
ARC_STSL_ [%]	Mean	65.3	70.9	76.1	71.7	70.8	67.3	71.6	61.1	67.2	86.5	74.2
	SD	10.7	13.8	33.8	-	11.3	13.4	12.8	11.3	11.5	8.3	1.1
	p	0.0274		0.1526								
ARC_ACSL_ [%]*	Mean	7.9	10.2	9.4	-	10.3	12.6	-	10.5	8.5	-	-
	SD	8.2	7	-	-	1	8.5	-	8.3	7.3	-	-
	p	0.2947		0.0489^†^								
ARC [%]	Mean	68.6	77.6	80.8	71.7	81.0	78.1	71.6	67.6	70.8	86.5	86.6
	SD	10.5	12.5	27.2	-	12.3	14.3	12.8	11.3	10.8	8.3	11.2
	p	0.00002		0.0966								

*When ACSL present.

^†^No differences in post-hoc test.

### Ambit occupation coefficient (AOC)

The value of the AOC was 61.2% ±10%. The ambit occupation coefficient for the STSL was 56.8% ±9.9% and for the ACSL 9.1% ±7.5%. Those parameters did not correlate with each other. However, there was a significant correlation between the AOC and SN passage area (R = -0.5609; p < 0.0001). Also, with respect to STSL type, correlation with the AOC was stronger in fan-shaped STSLs (R = 0.7331; p < 0.0001) than the band-shaped type (R = 0.6497; p < 0.0001). Also AOC_ACSL_ significantly correlated with SN passage area (R = -0.2658; p = 0.0082) but not as strongly as in the case of the AOC _STSL_ (R = -0.5609; p < 0.0001). Differences in ambit parameters between STSL and SSN types are presented in Table 2.

**Table 2:** **Characteristic of AOC associated parameters according to STSL and SSN types**

Parameters of area reduction		STSL		SSN type								
		Fan-type	Band-type	IA	IB	IC	II	IIIA	IIIB	IIIC	IV	V
Number		56	44	2	1	2	11	6	13	59	3	3
amSSN [mm]	Mean	31.5	28.2	34.1	47	35.1	28.65	30.18	33.05	29.19	36.5	19.23
	SD	5.95	5.65	4.1	-	0.85	5.64	5.42	7.7	4.89	1.8	7.64
	p	0.0061		0.0028^†^								
AOC_STSL_ [%]	Mean	55.5	58.3	59.7	60.4	59.2	55.3	61.1	52.2	56.7	67.1	64.5
	SD	7.9	11.9	21.4	-	2.2	10.8	10.4	8.7	10.1	1.6	1.7
	p	0.1797		0.0746								
AOC_ACSL_ [%]*	Mean	4.4	4.6	7.5	6.4	8.6	8.6	8.3	5.2	3.4	-	-
	SD	7.7	5.9	6.3	-	4.2	7.1	10.9	5.5	6.8	-	-
	p	0.8707		0.1491								
AOC [%]	Mean	59.9	62.9	67.3	66.8	67.8	63.9	69.5	57.4	60.1	67.1	64.5
	SD	11.6	12.6	27.7	-	2.0	13.9	18.2	11.1	11.8	1.6	1.7
	p	0.2331		0.0059^†^								

*When ACSL present.

^†^No differences in post-hoc test.

## Discussion

Structures surrounding the SN make it vulnerable to injury and compression by many different mechanisms. Direct compression caused by a mass in the SSN region (e.g. ganglion/labral cyst, tumour) [10, 11], repetitive irritation in overhead activities caused by the “sling effect” [7], continuous nerve traction following a rotator cuff tear [12, 13] or an inflammation process (e.g.viral neuritis) [14] can all lead to SN neuropathy. Anatomical variations of structures creating the osteo-fibrous tunnel for the SN can increase the risk of this pathology by constricting the nerve passage. Although it has been hypothesized that SN neuropathy is more likely to occur in patients with a narrow, V-shaped SSN, no direct correlation between SSN type and SN injury has been confirmed. Based on a cadaveric study, Ürgüden et al. [15] suggest that Rangachery type IV and V of the SSN may increase the risk of iatrogenic SN injury during rotator cuff tear repair, but no clinical data supports this theory. Furthermore, as shown in this study, the area of the SN passage does not differ between any particular type of SSN. Hence, it seems to be more important to evaluate the area for SN passage than just the type of SSN [10].

However, it is difficult to evaluate this area by means of imaging modalities. Magnetic resonance imaging reveals morphological changes in denervated muscles such as muscle oedema, muscle atrophy or fatty changes, but is limited with regard to the aetiology of nerve entrapment when no mass or cyst is affecting the nerve [12, 16, 17]. Furthermore, due to the position of the scapula, which is tilted anteriorly and rotated internally, standard shoulder MRI examination protocol does not allow for proper visualisation of the nerve passage. Even with an adjusted protocol, the exact evaluation of such a small structure might be challenging. Although 3-Tesla scanners support better resolution [18], 1.5-Tesla scanners are still more common, and the application of a higher magnetic field does not guarantee that the suprascapular ligament, which is usually 1-2mm thick, will be visualised in any of the scans.

The present study uses the newest classifications for both the SSN [9] and STSL [6]. This approach is simple, reproducible, and based on specific geometrical measurements that clearly distinguish each type, which is not the case for many existing schemes.

The bony border of the SSN can be visualised by classical radiography [12] or by CT scan [19]. However, no certain information about the STSL can be obtained, even in the case of patients with complete STSL ossification. The frequency of complete calcification varies between populations: It has been reported in 5.5% - 12.5% of cases [5, 20]. Although STSL ossification is a well-known risk factor of SN neuropathy [10], its presence was not included in this study as these parameters focus on the area of the nerve passage. Since the ossified ligament is an independent risk factor, its presence should always arouse suspicion of nerve irritation not pending on the tunnel area.

The majority of STSLs are non-ossified, and these can be visualised and evaluated by means of sonography [21–23]. According to the presented data, recognition of STSL type can have clinical implications, because the SN passage area is known to be significantly decreased in the case of band-shaped STSL types. Although the anterior coracoscapular ligament has also been suggested as a risk factor of SN neuropathy, only ARC_STSL_ and ARC, but not ARC_ACSL_ were found to be significantly increased in band-shaped STSLs. Furthermore, an inverse correlation exists between ARC_STSL_ and the ARC_ACSL_ but no correlation between AOC_STSL_ and the AOC_ACSL_. All the above suggest that the size of the ACSL relies to some extent on the size of the STSL, however, the STSL is the main factor constricting the SN passage area.

The proposed parameters have some limitations. Contrary to the ARC, calculation of the AOC does not require tools for area evaluation but only measurement of curves along the border of the SSN. However, this parameter does not take into account the curved inferior (STSL) or superior (ACSL) borders of the ligaments. Thus, AOC measurement can overestimate the predicted risk in ligaments with distinctly curved borders. On the other hand, the SSN ambit below the ACSL is not included in the formula, which may cause the risk to be underestimated.

Another factor that can bias the accuracy of the proposed parameters concerns the presence of an artery in the osteo-fibrous tunnel that usually houses only the nerve and vein. Anomalies of artery alignment were observed in 4% of analysed cases. This rate is higher than the 2.5% reported by Tubbs et al. [20] and 3% by Reineck et al. [24], but lower than the 10.9% reported by Yang et al. [25]. It was suggested that when the artery neighbours the nerve directly, it might exert blood pressure on the more fragile nerve, causing microtrauma to the nerve, ultimately resulting in neuropathy [20]. Also the varix of the suprascapular vein, which has only been reported at the level of the spinoglenoid notch, might reduce the free area for the nerve. However, due to difficulties in clinical evaluation of those small vessels and their unconfirmed aetiological status, they were not considered when calculating the parameters.

## Conclusion

Both the ARC and the AOC are well correlated with the SN passage area, and so might be used in the evaluation of the risk of SN neuropathy, without direct visualisation of the nerve passage. Furthermore, the ARC is characterised by lower standard deviation with respect to the SN passage area. Hence, due to the difficulty of passage area evaluation, and the limitations associated with AOC, ARC seems to be the most relevant and reliable parameter for evaluating the constellation of anatomical risk factors of SN neuropathy. The usefulness of proposed parameters should be further tested under clinical conditions.

## Authors’ original submitted files for images

Below are the links to the authors’ original submitted files for images. Authors’ original file for figure 1 Authors’ original file for figure 2 Authors’ original file for figure 3 Authors’ original file for figure 4
