# Supplementary figures and images for: New parameters describing morphological variations in the suprascapular notch region as potential predictors of suprascapular nerve entrapment
Source: BMC Musculoskelet Disord. 2014 Nov 25;15:396. doi: 10.1186/1471-2474-15-396 (PMC4256739; doi:10.1186/1471-2474-15-396)

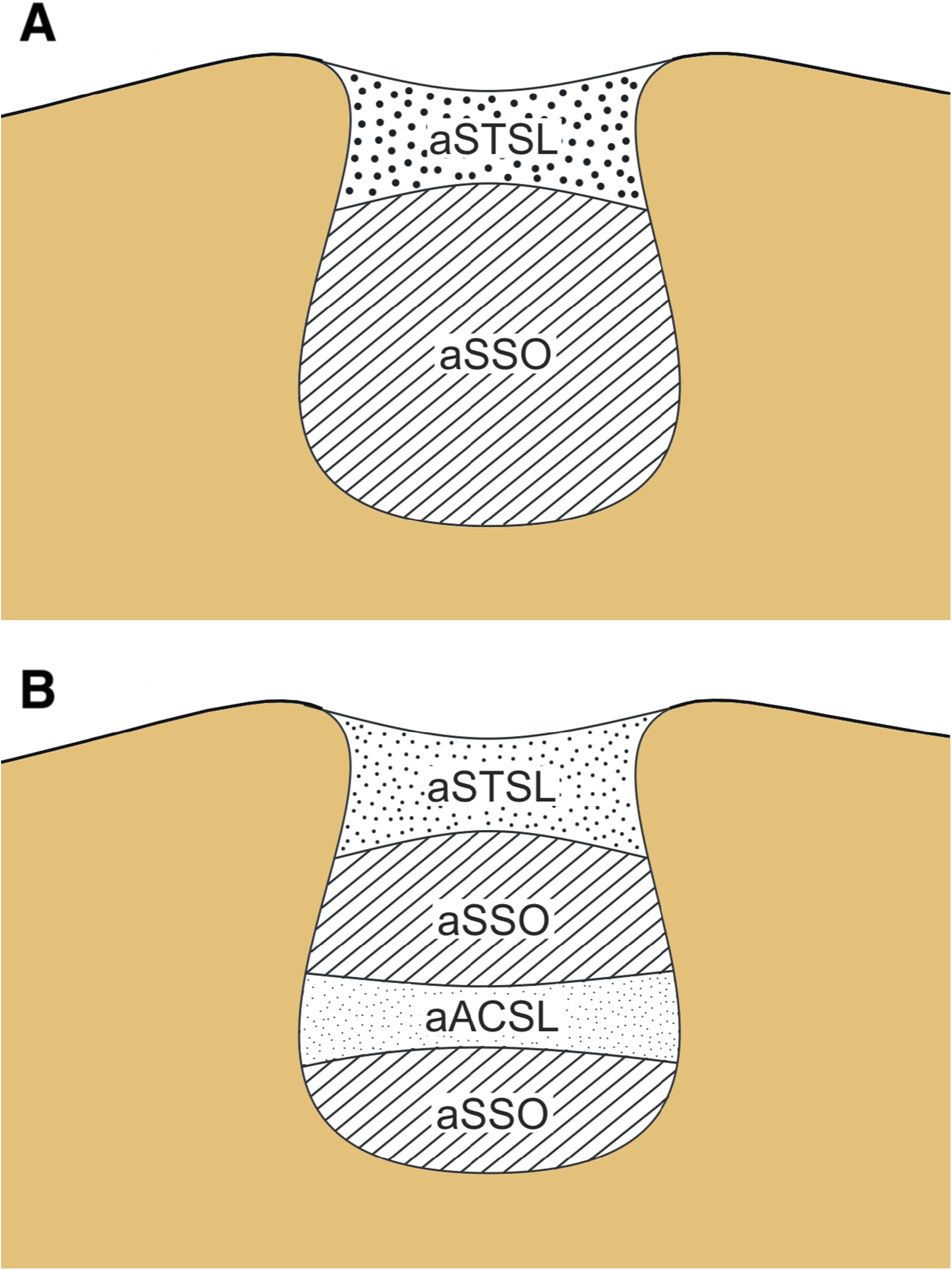

Supplement: Supplementary file 1 — Authors’ original file for figure 1 [file 12891_2013_2334_MOESM1_ESM.tif]

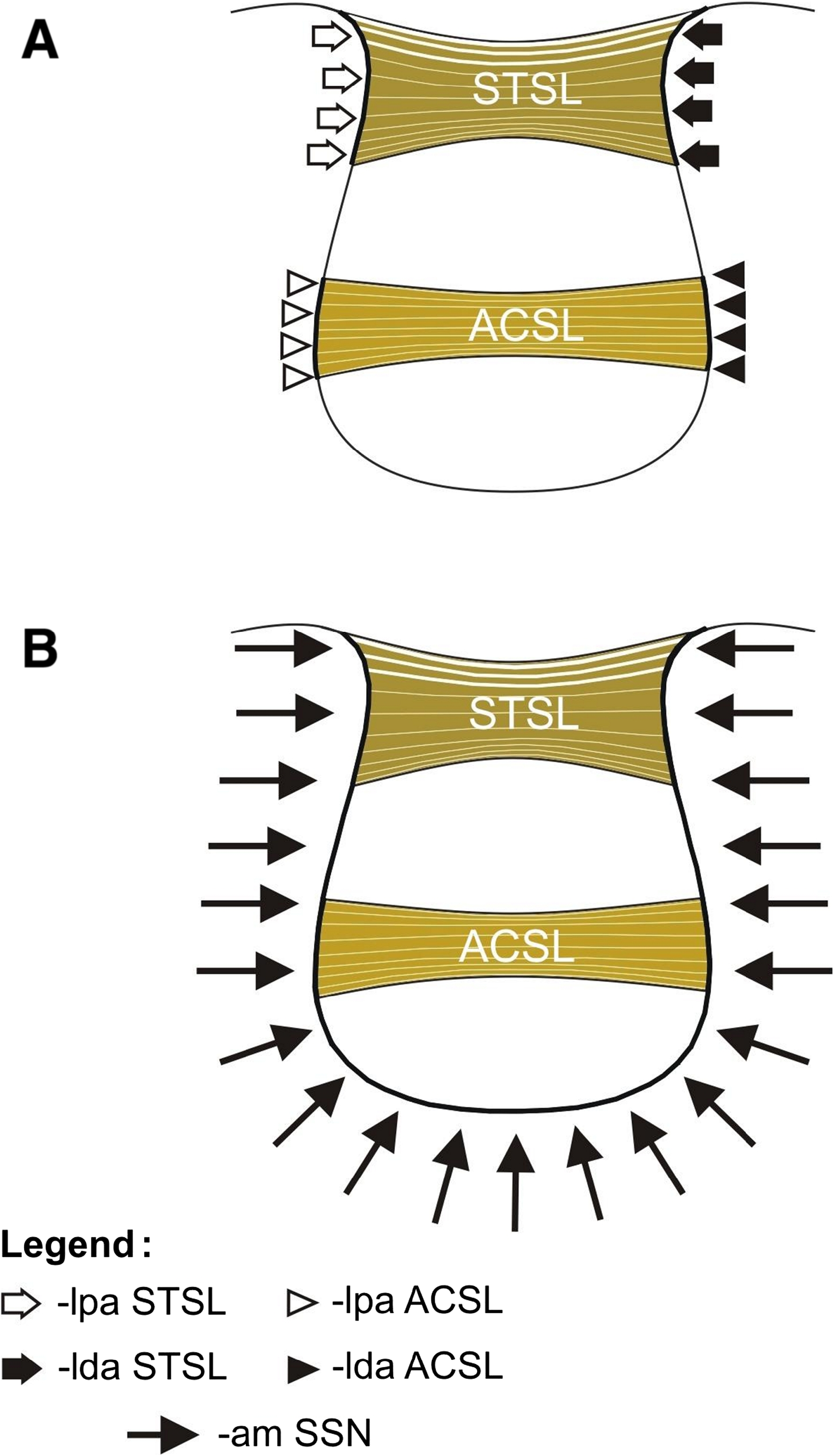

Supplement: Supplementary file 2 — Authors’ original file for figure 2 [file 12891_2013_2334_MOESM2_ESM.tif]

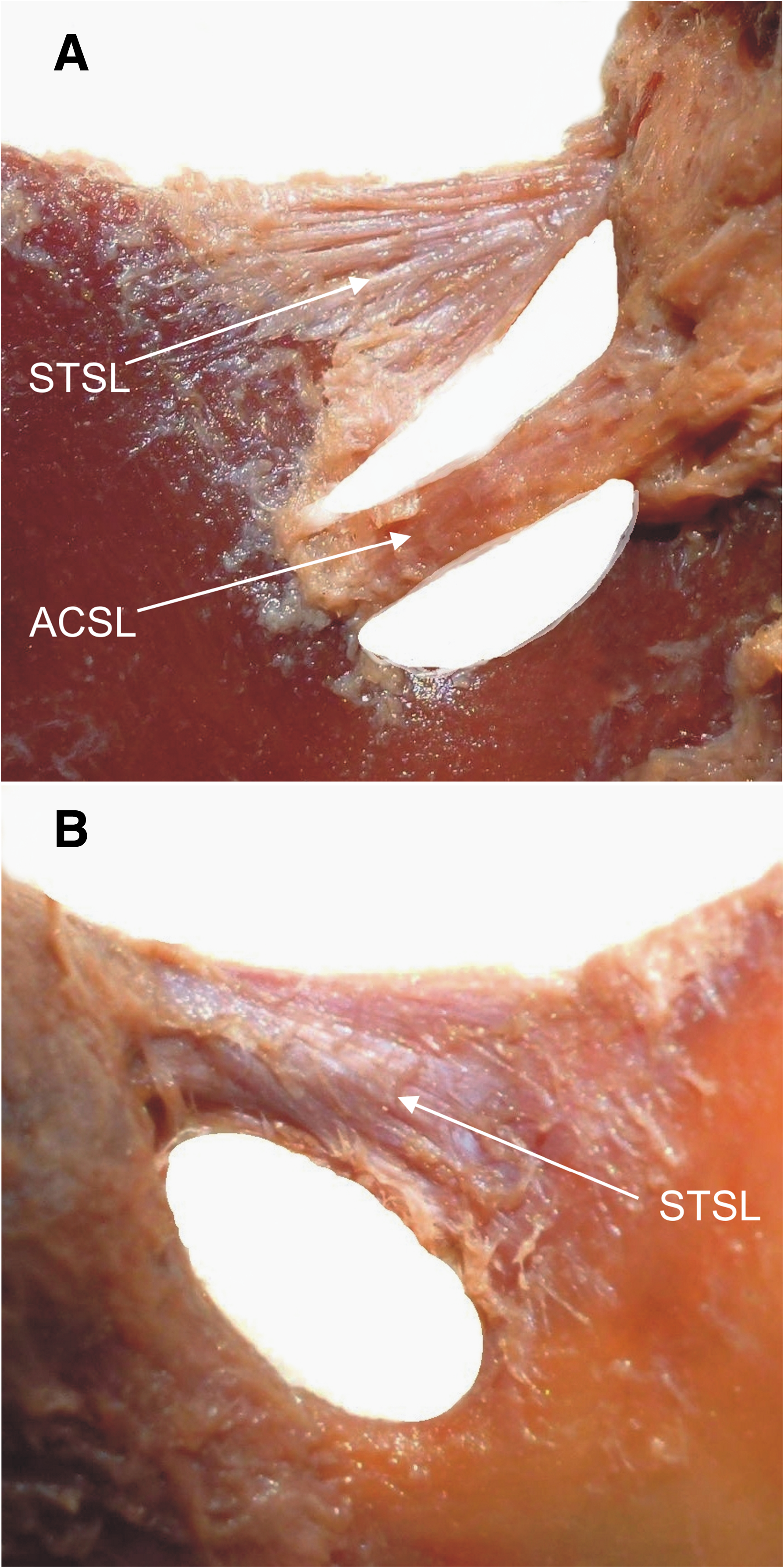

Supplement: Supplementary file 3 — Authors’ original file for figure 3 [file 12891_2013_2334_MOESM3_ESM.tif]

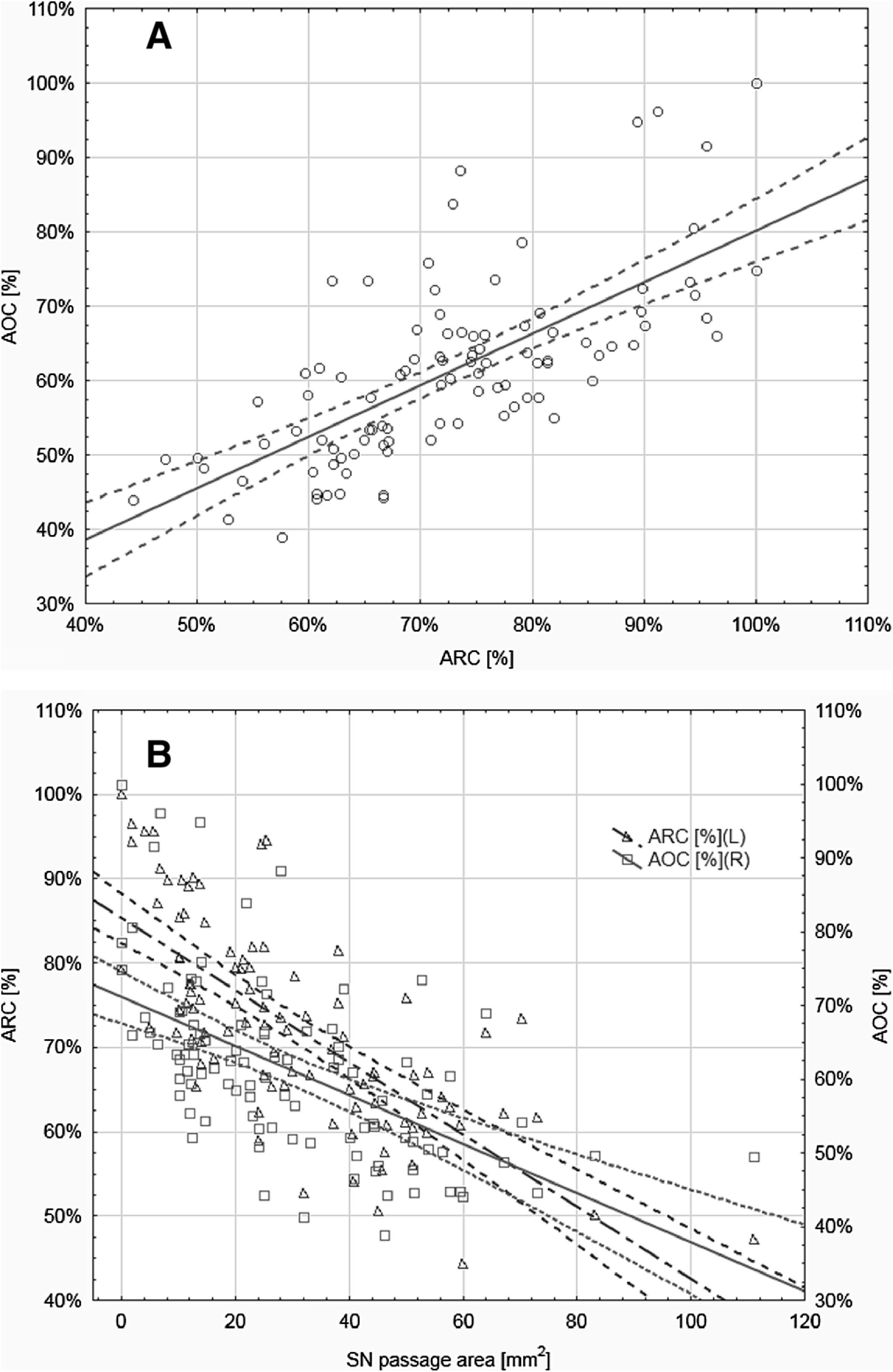

Supplement: Supplementary file 4 — Authors’ original file for figure 4 [file 12891_2013_2334_MOESM4_ESM.tif]
